# Field Test of a Bioelectrochemical Membrane‐Less Reactor for Chlorinated Aliphatic Hydrocarbon and Nitrate Removal from a Contaminated Groundwater

**DOI:** 10.1002/cplu.202400683

**Published:** 2025-06-16

**Authors:** Geremia Sassetto, Maria Presutti, Agnese Lai, Giulia Simonetti, Laura Lorini, Marco Petrangeli Papini, Marco Zeppilli

**Affiliations:** ^1^ Department of Chemistry University of Rome Sapienza Piazzale Aldo Moro 5 00185 Rome Italy; ^2^ Research Center for Applied Sciences to the Safeguard of Environment and Cultural Heritage (CIABC) University of Rome Sapienza Piazzale Aldo Moro 5 00185 Rome Italy

**Keywords:** bioremediation, microbial electrolysis cells, oxidative dechlorination, permeable reactive barriers, reductive dechlorination

## Abstract

This study uses a membrane‐less reactor to explore the bioelectrochemical remediation of real contaminated groundwater from chlorinated aliphatic hydrocarbons (CAHs) and nitrates. The research focuses on testing a column‐type bioelectrochemical reactor to stimulate in situ degradation of contaminants through the supply of electrons by a graphite granules biocathode. After a preliminary laboratory characterization and operation with a synthetic feeding solution, a field test is conducted in a real contaminated site, where the reactor demonstrates effective degradation of CAHs and inorganic anions. Notably, the cathodic potential promotes the reductive dechlorination of chlorinated species. Simultaneously, nitrate reduction, sulfate reduction, and methanogenesis occurr, influencing the overall coulombic efficiency of the process. The use of real groundwater, compared to the synthetic medium, significantly decreases the coulombic efficiency of reductive dechlorination, dropping from 2.43% to 0.01%. Concentration profiles along the bioelectrochemical reactor allow for a deeper description of the reductive dechlorination rate at different flow rates, as well as increase the knowledge about reduction and oxidation mechanisms. Scaling up the technology presents several challenges, including the optimization of coulombic efficiency and the management of competing microbial metabolisms. The study provides a valuable contribution toward advancing bioelectrochemical technologies for the bioremediation of complex contaminated sites.

## Graphical abstract

This study evaluates a membrane‐less bioelectrochemical reactor in a real groundwater remediation scenario, targeting chlorinated aliphatic hydrocarbons and nitrate contamination. Electron supply via graphite granules biocathode stimulates reductive dechlorination and nitrate reduction, despite competing processes like sulfate reduction and methanogenesis. Field results reveal effective contaminant degradation, highlighting critical considerations for scaling up bioelectrochemical remediation technologies at complex environmental sites.

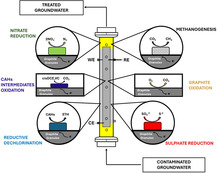

## 1 Introduction

Chlorinated aliphatic hydrocarbons (CAHs) are ubiquitous contaminants that pose a significant threat to groundwater resources due to their persistence in the environment and risks to human health. These compounds, often classified as dense nonaqueous phase liquids (DNAPLs), are frequently present in groundwater due to their inappropriate use and disposal for several industrial activities. The nature of DNAPLs leads them to infiltrate deep subsurface layers, generating contamination plumes that can persist for decades. Indeed, remediation of CAHs‐contaminated groundwater constantly represents a significant challenge, requiring innovative and energy‐efficient solutions.^[^ ^1^ ^]^

Currently, in situ bioremediation represents one of the most promising remediation strategies to address CAHs contamination, since it exploits indigenous microorganisms' metabolic potential to degrade contaminants into harmless products. In this regard, biological reductive dechlorination (BRD) is a well‐documented anaerobic bioremediation process in which bacteria utilize CAHs as electron acceptors, breaking them down into less‐chlorinated compounds and ultimately into nontoxic ethene (ETH).^[^ ^2^ ^]^ Several bacterial species, including the genus *Dehalococcoides*, can perform BRD and complete the conversion of CAHs to ethene.^[^ ^3^ ^]^

**Figure 1:**
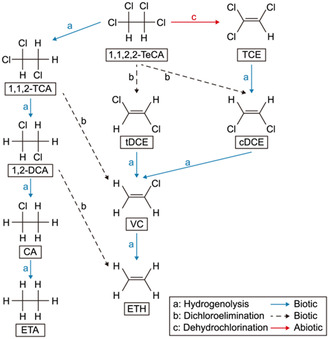
Anaerobic degradation pathways for chloroethanes and chloroethenes. Figure Reproduced with the permission of Elsevier.^[^ ^2^ ^]^

Reductive dechlorination of CAHs, such as 1,1,2,2‐tetrachloroethane (TeCA) and trichloroethylene (TCE), occurs through stepwise biological processes primarily driven by dehalorespiring microorganisms. Two key mechanisms dominate these reactions: hydrogenolysis and dichloroelimination. Hydrogenolysis involves the sequential replacement of chlorine atoms with hydrogen atoms, progressively reducing the chlorination degree of the compound. For example, TeCA undergoes hydrogenolysis to form trichloroethane (TCA), which is further reduced to dichloroethane (DCA) and ultimately ethane (ETA). Dichloroelimination, on the other hand, removes two chlorine atoms simultaneously, resulting in the formation of unsaturated intermediates such as cis‐ and trans‐dichloroethylene (DCE). These intermediates are further reduced to vinyl chloride (VC), and eventually, to nontoxic end‐products like ethene (ETH) and ethane (ETA) – **Figure** **1**. The complete dechlorination pathway depends on environmental conditions, electron donors, and the presence of specialized microorganisms, such as *Dehalococcoides mccartyi*, capable of catalyzing these critical steps.^[^ ^2^ ^]^

On the other hand, the actual efficiency of BRD is closely linked to the availability of electron donors, mainly molecular hydrogen (H_2_), which is often limited in contaminated aquifers. To improve BRD activity, enhanced natural attenuation is commonly employed by supplying fermentable organic substrates that release H_2_ as a by‐product of their degradation. Despite innovative biobased and more sustainable electron donors^[^ ^4^, ^5^, ^6^ ^]^ (i.e., polyhydroxyalkanoates, PHA) have been utilized as electron donor for reductive dechlorination stimulation, their injection can cause undesired side effects, since the abundance of organic substrates may trigger additional reactions within the aquifer, ultimately damaging the water quality.^[^ ^1^, ^7^ ^]^ In addition, competing microbial processes, such as sulfate reduction and nitrate reduction, can compete for electron donors and hinder BRD activity.^[^ ^8^ ^]^

Bioelectrochemical systems (BES) have emerged in the last decades as an innovative approach for the remediation of CAHs‐contaminated groundwater. To overcome the limitations of conventional methods, BES utilize electrodes to deliver a controlled flow of electrons to microorganisms, facilitating a continuous and sustainable supply of reducing power for BRD. In fact, in a typical BES for CAHs remediation, a polarized cathode acts as an electron donor for CAHs‐reducing bacteria, enabling the BRD process without adding organic substrates. This particular ability of BESs to precisely control the electron supply by modulating the potential of the cathode offers a significant advantage over traditional enhanced natural attenuation methods, minimizing potential side effects and improving the overall efficiency of the remediation process.^[^ ^9^ ^]^ Several laboratory‐scale studies have demonstrated the potential of BES in achieving high CAHs removal rates, in some cases reaching the complete mineralization of contaminants into harmless end products. Despite these promising results, field implementation of BES for groundwater remediation remains relatively limited, and the evaluation of the long‐term effectiveness of this innovative technology, under complex real‐world conditions, results crucial for its wider adoption.

In this view, several groups have successfully demonstrated BES remediation on a pilot‐scale.^[^ ^9^, ^10^, ^11^ ^]^ Scaling up BES from the laboratory to pilot‐scale is a highly multidisciplinary effort which includes electrochemical, biological, and environmental knowledge. It requires detailed knowledge of all relevant operating parameters, particularly those that benchmark BES against conventional technologies. For instance, unlike BESs used for wastewater treatment, in situ applications for groundwater remediation demand more adaptable and simplified BES configurations to adapt the process to varying depths and heterogeneous physical or chemical conditions usually present in the subsurface. Additionally, groundwater typically contains multiple contaminants, necessitating an integrated remediation approach. However, most studies have focused on removing either a single contaminant or mixed contaminants of the same type (electron donors or acceptors), highlighting the need for further research to address coexisting contaminants of different types.^[^ ^11^ ^]^ In this study, a new designs column‐type BES reactor was tested for enhanced biodegradation of CAHs in real groundwater polarizing the cathodic chamber at different levels. The technology presented here is founded upon the concept of a bioelectrochemical reactor working as a permeable reactive barrier (PRB). The primary appeal of PBR‐like reactors has been the low power cost and the potential to address contaminants that might otherwise be difficult to treat with existing technologies. This research illustrates a novel electrolytic reactive barrier technology using a PBR‐like bioelectrochemical reactor that treats pollutants in groundwater. Moreover, several laboratory‐scale studies have supported field efforts. In detail, contaminants were pumped through the PBR‐like reactor by a peristaltic pump working at 2.5 Ld^−1^, the same natural groundwater flow. Within the reactor, contaminants were degraded as they passed through graphite electrodes charged at −0.65 V versus SHE (standard hydrogen electrode) cathodic potential applied. In these conditions, contaminants were exposed to bioelectrochemical reduction. The present study aimed to test the feasibility of the bioelectrochemical remediation approach in these realistic conditions. This test was conducted in a contaminated area located 14 km northwest of Milan, Italy. Results from this study highlight the capability of the bioelectrochemical process to boost dechlorination and anion reduction in groundwater simply by tuning the polarization of a simple and effective reactor design.

## 2 Experimental Section

### 2.1 Site Description

A former chemical facility mainly involved in the production of synthetic dyes is the source of a chlorinated solvent plume that extends over ≈0.4 km^2^.^[^ ^12^, ^13^, ^14^ ^]^ The plume has originated from the leakage of an underground storage tank located ≈20 m outside the industrial area, located 2–6 m below ground surface (b.g.s.). Both a shallow (from 5 to 12 m b.g.s.) and a deep (from 15 to 40 m b.g.s.) aquifer underlying the industrial site have been impacted by the contamination with the formation of DNAPL pools acting as long‐term sources of contamination. In 1982, local authorities undertook an emergency containment action, which consisted of the lateral and superficial isolation of the storage tank.

### 2.2 Aquifer Sample Collection

Twenty‐five liters of groundwater were collected from the deep aquifer of the contaminated site in a sterile collapsible Tedlar bag. The location for groundwater sampling was chosen based on the outcome of preliminary chemical analyses, which indicated the presence in the groundwater of biodegradation products (i.e., vinyl chloride ‐ VC and cis‐dichloroethylene ‐ cis‐DCE), suggesting the presence of native dechlorinating microbial populations.

### 2.3 Water Characterization

The deeper aquifer (from 15 to 50 m b.g.s.) used had been qualitatively and quantitatively precharacterized in the chlorinated solvent and inorganic anions. **Table** **1**, **2** summarizes groundwater characterization results. The deeper aquifer was primarily contaminated by trichloroethylene (TCE). Groundwater characterization revealed the presence of perchloroethylene (PCE), cis‐DCE, trans‐DCE, 1,1,1‐Trichloroethane (TCA), and 1,2‐Dichloroethane (1,2‐DCA). 1,1,2‐TCA, VC, ETH, and ethane (ETA), which are also known to be 1,1,2,2‐Tetrachloroethane (1,1,2,2‐TeCA) and TCE dechlorination byproducts, were not detected in groundwater samples. CAHs showed a high concentration, and the highest CAH concentration measured was 0.918 mgL^−1^ of cis‐DCE. The determination of cis‐DCE suggested the presence of an indigenous dechlorinating activity in the contaminated site, probably due to the biological hydrogenolysis of TCE and dichloroelimination of 1,1,2,2‐TeCA. The presence of different chlorinated intermediates is consistent with the historical contamination of the site, detected for an extremely long time (over 20 years).^[^ ^15^ ^]^ Inorganic anions characterization (Table 2) indicates a 39.0 ± 0.8 and 100.0 ± 3.0 mgL^−1^ concentration, respectively. As already described,^[^ ^5^ ^]^ sulfate and nitrate compete for the reducing power, being additional electron acceptors competing with the reductive dechlorination of chlorinated solvents. Furthermore, field parameters were measured in conjunction with collecting water samples for analysis of chlorinated compounds. Field parameters included pH, chemical oxygen demand (COD), total organic carbon (TOC), inorganic carbon (IC), volatile suspended solids, fixed suspended solids, total suspended solids, and conductivity. Results for field‐measured parameters are presented in Table 2. The groundwater conductivity was similar to synthetic medium (which was 4.6 ± 0.2 mScm^−1^). The groundwater pH resulted in the.^[^ ^16^ ^]^ The organic carbon was negligible in groundwater, while the COD was below the analytical detection limit (i.e., <25 mgL^−1^).

**Table 1:** Groundwater characterization and relative contamination threshold value established by italian legislation (annex 5, part IV, title V of legislative decree 152/2006).

	Average Value	Standard deviation	CSC [mgL^−1^]
1,2 DCA	0.380 mgL^−1^	±0.040	3
Cis‐DCE	0.370 mgL^−1^	±0.040	60
Trans‐DCE	0.170 mgL^−1^	±0.040	
1,1,1 TCA	0.050 mgL^−1^	±0.001	
TCE	0.270 mgL^−1^	±0.030	1.5
PCE	0.014 mgL^−1^	±0.002	1.2
1,1,2,2‐TeCA	0.100 mgL^−1^	±0.001	0.5
Chloride	29.1 mgL^−1^	±0.8	
Nitrate	39.0 mgL^−1^	±0.8	
Sulfate	100 mgL^−1^	±3	250
pH	6.7	±0.3	
COD	<25 mgL^−1^		
TC	56.9 mgL^−1^	±0.1	
IC	42.1 mgL^−1^	±0.1	
Conductivity	2.2 mScm^−1^	±0.3	

**Table 2:** Parameters of pilot‐scale reactor.

Parameter	Cathode	Anode	Extremities
Volume [cm^3^]	4074	24 022	1020 785
Graphite (g)	3641	1641	
Nonreactive material (g)			1507 1179
Effective volume [cm^3^]	2037	1201	

### 2.4 Bioelectrochemical Reactor Set‐up

**Figure 2:**
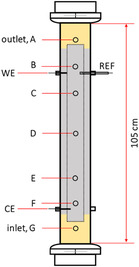
Scheme of the bioelectrochemical PBR‐like reactor.

The bioelectrochemical reactor consisted of a borosilicate glass column, with a height of 105 cm and an internal diameter of 10 cm, giving a geometric empty volume of 8242 cm^3^. To characterize the spatial reactor performance, seven sampling ports were present along the column (named from A to G) (**Figure** **2**). The sampling ports were placed respectively at 7 (port A), 20 (port B), 32 (port C), 53 (port D), 72 (port E), 85 (port F), and 98 cm (port G) from the top of the column. Additional ports have been utilized for the placement of current collectors and the Ag/AgCl in saturated KCl electrode (E°' = 199 mV vs SHE) reference electrode used to control the cathodic potential. Port G was used to feed the contaminated solution from the bottom, while port A of the reactor was used for the effluent draw. Graphite granules (Faima srl, Milano, Italy) with an average diameter of 2 mm were used as electrodic material for the anodic and cathodic chambers. Before use, the graphite granules were pretreated with HCl and NaOH (1 M) solutions and dried at 100 °C before their use in the reactor. The anodic and cathodic electrodes had been placed in a concentric configuration in which the cathodic chamber constituted the outer part of the cylinder while the anodic chamber was placed in the inner part of the reactor, as already described in previous works.^[^ ^17^ ^]^ The anode chamber was divided from the cathodic chamber using a plastic cylinder made of a PE‐HD mesh with a 1.2 × 1.2 mm pore size. After filling with graphite granules (bed porosity of almost 50%), the liquid phase volume at the cathode and the anode was reduced to 2 L each (Table 2).

The cathodic and anodic electrical connectors were two graphite rods (300 mm × ID 8 mm) connected in series by titanium wire (5 cm), for each compartment. The cathodic volume was 4074 cm^3^, and the anodic volume was 2402 cm^3^. The column extremities were filled with 10 cm^3^ silica beads to prevent the site soils from infiltrating and clogging the electrodes. The anodic compartment was inserted into the nonconductive layer to 25 mm at each end.^[^ ^16^ ^]^ The cathode (working electrode, WE), anode (counter electrode, CE), and reference electrode were connected to an Amel Model 551 (Milan, Italy) potentiostat, which was used to control the cathodic potential at the value of −650 mV versus SHE.

The influent was fed by a peristaltic pump, and it was maintained in a collapsible Tedlar bag (25 L) that contained no headspace. The effluent was collected in a plastic tank with an active carbon trap. Before the start‐up, the cathode chamber was inoculated with 0.7 L of a TCE‐to‐ethene dechlorinating culture composed of 75% of *Dehalococcoides* *McCarty*. Raw data of the consortium's 16S rRNA gene amplicon sequencing are available at the DDBJ/ENA/GenBank under the BioProject PRJNA705054 (SRA: SRX10172732).

### 2.5 Bioelectrochemical Reactor Operating Conditions

#### 2.5.1 Preliminary Laboratory‐Scale Studies

A Laboratory test was conducted in two phases. The first phase included the system optimization through 8 h batch operation cycles and the fluid dynamic characterization of the reactor. To characterize the fluid dynamic behavior of the cathode compartment of the reactor, a tracer test was carried out, as previous studies described using.^[^ ^17^ ^]^ The second phase of the laboratory test was conducted in continuous flow using an organic load rate of 2.3 μmolL^−1^ h^−1^ of TCE. Throughout the entire operational period, the cathodic potential applied was −650 mV versus SHE. The cathodic potential value was chosen according to the electrons necessary to complete the RD and anions reduction through the hydrogen evolution reaction (the H_2_ standard reduction potential is −414 mV at pH 7). The bioelectrochemical reactor was fed with an oxygen‐free anaerobic medium. The medium contained (in gL^−1^): NH_4_Cl, 0.5; MgCl_2_, 3; 6H_2_O, 0.1; K_2_HPO_4_, 0.4; MgCl_2_, 3; 2H_2_O, 0.05; 2 mLL^−1^ of a metal solution;^[^ ^18^ ^]^ and 2 mLL^−1^^[^ ^19^, ^20^, ^21^ ^]^ of vitamin solution. The vitamin solution insertion in the synthetic medium was addressed to improve the growth of the dechlorination biofilm and enhance microbial activity during laboratory tests. During the field test, no vitamins were added. The electrical conductivity of the medium was 4.8 mScm^−1^, hence within the range of values typically reported for highly contaminated groundwater (i.e., 0.67 to 7.98 mScm^−1^). The pH of the medium was maintained at values between 7 and 7.5 with a NaHCO_3_ solution (10% w/v). The temperature inside the reactor and in the sampling cells was maintained at room temperature, ≈20 ± 5 °C.

#### 2.5.2 Fluid Dynamic Characterization

**Figure 3:**
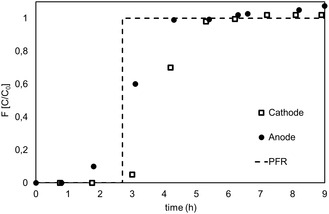
The cathodic and anodic experimental residence time distribution curve F(t) and the theoretical plug flow (PFR) RTD curves in reactor output (sampling port F).

The simplest and most direct way of finding the E and F curves was to use a nonreactive tracer by a step experiment. The tracer test showed that the reactor fluid dynamic behavior was like the plug‐flow model in reactor output collected by sampling port B at −650 mV versus SHE cathodic potential applied and 5.5 Ld^−1^ (Figure S1‐A, Supporting Information). By the curve F(t), the effective hydraulic residence time (t) was determined, and it was 16.1 h (Figure S1‐B, Supporting Information). The effective porosity of 0.53 was determined from the ratio between the theoretical (total empty reactor volume) and effective HRT. This value was consistent with previous analysis of the granules of graphite used as reactor filling. However, the porosity was estimated using the total reactor volume, indicating a cathode–anode nonseparation flow (**Figure** **3**). The overlapping shape and position of the cathode and anode F(t) curves were an additional confirmation (Figure 5). Moreover, the shape of the F curve depends on dispersion and the boundary conditions for the vessel. The resulting S‐shaped response curves were not symmetrical (Figure 5) which indicates a large deviation from the PF model.

#### 2.5.3 Field Pilot‐Scale Studies

The groundwater was used as the reactor feed to the continuous flow reactor at an influent flow rate of 2.5 Ld^−1^, corresponding to an HRT of 1.6 d in the cathodic compartment (relative to the empty volume). The working electrode (i.e., the cathodic chamber) was polarized at −650 versus SHE, for an overall period of about 75 days, to investigate the influence of cathodic potential applied on the degradation efficiency. The experiment was conducted with a thermal sleeve reactor to maintain a constant temperature of about 15 °C.

### 2.6 Analytical Procedures and Monitoring Protocol

Every 3 days, the inlet (port G) and outlet (port A) samples were collected in 10 mL glass vials and analyzed for chlorinated solvents and inorganic anions. Concentration profiles along the reactor were collected with the same procedure using the different ports along the height of the reactor. Before preparation, the bottles were sealed with Teflon‐faced butyl rubber stoppers and aluminum crimp caps and fluxed with N_2_/CO_2_ (70/30 % v/v) gas mixture. Vinyl chloride, ethene, ethane, and methane were quantified in headspace samples by using gas chromatography (GC) with a flame‐ionization detector (FID).^[^ ^22^ ^]^ Chloroethanes and chloroethenes were quantified by injecting 1 mL of vials headspace into a Dani GC 1000 (Contone, Switzerland) GC equipped with an FID detector (capillary TRB 264 column, length 75 m, Teknokroma (Spain); N_2_ carrier gas 14.5 mLmin^−1^; oven temperature from 80 °C to 210 °C; flame ionization detector temperature 260 °C). Hydrogen was analyzed in 500 μL headspace samples using GC with a thermal conductivity detector as described elsewhere.^[^ ^23^ ^]^ Standards for chlorinated compounds, ETH, ETA, CH_4_, and H_2_ were prepared by adding, with a gastight syringe, a known amount of each compound to a serum bottle with the same headspace‐to‐liquid ratio as the vial's sample.^[^ ^24^ ^]^ Concentrations for volatile compounds were expressed as total moles in the bottle divided by the liquid phase (i.e., nominal concentrations). Liquid samples (3 mL) were taken using sterile disposable plastic syringes, filtered (0.20 μm), and analyzed for nitrate, nitrite, sulfate, and chloride, by using ion chromatography (0.25 μL sample, Dionex DX‐100, Ionpac As9‐Sc column, conductivity detector). All the chemicals used were analytical grade.

### 2.7 Calculation

The average rate of reductive dechlorination was calculated from the measured concentrations of dechlorination intermediates from different dechlorinating pathways as

(1) $$r_{{\text{RD}}_{\text{tot}}}\left[\frac{\text{μeq}}{\text{Ld}}\right]=\frac{{\text{ΔC}}_{\text{liq}}\left(\text{1,1,2TCA}\right){\cdot 2+\text{ΔC}}_{\text{liq}}\left(\text{1,2DCA}\right){\cdot 4+\text{ΔC}}_{\text{liq}}\left(\text{cisDCE}\right){\cdot 2+\text{ΔC}}_{\text{liq}}\left(\text{transDCE}\right){\text{ ·2+ΔC}}_{\text{liq}}\left(\text{VC}\right){\text{ ·4+ΔC}}_{\text{liq}}\left(\text{ETH}\right){\text{ ·6+ΔC}}_{\text{liq}}\left(\text{ETA}\right)\text{ ·8}}{V_{c}}\text{ · Q}$$

where 2, 4, 6, and 8 are the number of moles of electrons required for the formation of 1 mole of each intermediate, taking into account dichloroelimination and hydrogenolysis of 1,1,2,2 tetrachloroethane (1,1,2,2‐TeCA) pathways; the ΔC_liq_ is the compound concentration differences (μmolL^−1^) between the reactor influent and sampling port; Q is the flow rate (Ld^−1^); and V_C_ is the empty volume of the cathode chamber relative to each sampling port (L).

Analogously, the methane production rate was calculated as

(2) $$r_{{\text{CH}}_{4}}\left[\frac{\text{μeq}}{\text{Ld}}\right]=\frac{{\text{ΔC}}_{\text{liq}}\left({\text{CH}}_{4}\right)\text{ ·8}}{V_{c}}\text{ ·Q}$$

where 8 is the number of moles of electrons required for the formation of 1 mol of methane, and the ΔC_liq_ is the different in‐sampling port methane concentrations (μmolL^−1^).

The rates of the reduction processes of the anions were calculated from the concentration change and converted in terms of μeqL^−1^ by assuming the complete conversion through the following half‐reactions
(3)

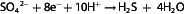

(4) $${\text{NO}}_{3^{-}}{\text{ + 5e}}^{‐}{\text{ + 6H}}^{+}{\to N}_{2}{\text{ + 3H}}_{2}O$$

(5) $$r_{\text{nitrate}}\left[\frac{\text{μeq}}{\text{Ld}}\right]=\frac{{\text{ΔC}}_{\text{liq}}\left({\text{NO}}_{3}^{‐}\right)\text{.5}}{V_{c}}\cdot Q$$

(6) $$r_{\text{sulphate}}\left[\frac{\text{μeq}}{\text{Ld}}\right]=\frac{{\text{ΔC}}_{\text{liq}}\left({\text{SO}}_{4}^{\text{2‐}}\right)\cdot \text{8}}{V_{c}}\cdot Q$$

where 5 and 8 are the number of moles of electrons required for the reduction of 1 mole of each inorganic anions, and the ΔC_liq_ are the cathodic concentrations influent and sampling port difference (μmolL^−1^).

The coulombic efficiency (%) was calculated as the ratio of electric current due to the reductive reaction and the current flowing across the system according to the following equation

(7) $$\text{CE }\left[\%\right]=\frac{r\cdot \text{F }}{I}\cdot \text{100}$$

where *r* was the reaction rate $\left(\frac{\text{μeq}}{\text{Ld}}\right)$, F was Faraday's constant (96 485 Cmol electrons^−1^), and I was the electric current $\left(\frac{\text{μeq}}{\text{Ld}}\right)$.

The CAHs removal percentage was determined as follows

(8) $$\text{CAHs removal }\left[\%\right]=\frac{{\text{ΣCAHs}}_{\text{in}}{\text{ ‐ ΣCAHs}}_{\text{sampling port}}\;}{{\text{ΣCAHs}}_{\text{in}}}\cdot \text{100}$$

## 3 Results and Discussion

### 3.1 Laboratory Test ‐ Bioelectrochemical Reactor Start‐up

**Figure 4:**
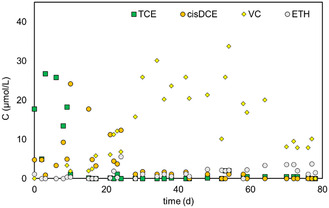
CAHs, ETH (A) concentration profile as a function of time during the 5.5 Ld^−1^ operating period.

After the preliminary fluid dynamic characterization, the bioelectrochemical reactor was run in the continuous mode with the mineral medium as feed, at an initial TCE contamination of 46.7 ± 32.0 μM and a 5.5 Ld^−1^ flow rate for more than 49 d. The cathodic potential applied was −650 mV versus SHE. Following inoculation and acclimation, the electrode different potentials anode potential of the pilot‐scale BES decreased from −1.0 to −1.8 V (Figure S2, Supporting Information), and it became stable in 6 d. The cathodic current gradually increased to about 32 mA during the initial period, indicating that the electrochemically active microorganisms justified the activation of microbial metabolisms. During the start‐up, the system was well buffered in the presence of NaHCO_3_ in the mineral medium, and the pH was maintained steadily at about pH 7.0. The time course of CAHs concentrations (**Figure** **4**) and methane (Figure S3, Supporting Information) in the effluents of the bioelectrochemical reactor, corresponding to the sampling port A, is reported in Figure 4.

**Figure 5:**
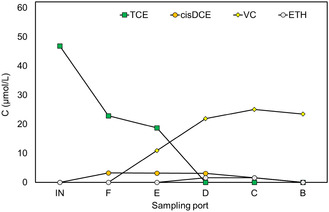
CAHs concentration profile in function on column length at 5.5 Ld^−1^.

After an acclimatization period of 28 days, the reactor reached a steady state. During this period, the typical biological dechlorination concentration profile was performed, and the concentrations of cis‐DCE, VC, and ethene increased before reaching a nearly stable value (1.17 ± 0.23, 23.4 ± 1.8, and 0.57 ± 0.12 μmolL^−1^, respectively). This evidence suggested the presence of the *Dehalococcoides* microorganisms, which were necessarily involved in RD steps beyond cDCE, in the cathodic compartment.^[^ ^25^ ^]^ After this period, VC was the main product of reductive dechlorination with lower amounts of ETH (0.57 ± 0.12 μmolL^−1^). The methane concentration increased from 1 to 28 days; it became stable at 119 ± 17 μmolL^−1^. Methane is a typical by‐product obtained during the operation of bioelectrochemical reactors under anaerobic conditions.^[^ ^26^ ^]^ A typical concentration profile of chlorinated compounds (**Figure** **5**) and methane (Figure S4, Supporting Information) as a function of column length during a 5.5 Ld^−1^ operating period is reported.

**Figure 6:**
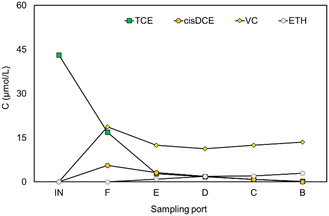
CAHs concentration profile in function on column length at 2.5 Ld^−1^.

The TCE dechlorination was observed in the column by the first sampling ports after the reactor inlet (F), and the RD rate increased along the column. At sampling port B (column outlet), VC was the major metabolite (93%), and only small amounts of cis‐DCE (5%) and ETH (2%), TCE was measured in the column effluent too (0.5%). At the outlet column, the TCE influent concentration was further reduced to 0.12 μmolL^−1^, resulting in a TCE rate removal of 30.6 μmolL^−1^ d^−1^. However, the RD performance remained stable after sampling port D (middle point) when the methane production was more evident with the higher concentration. To evaluate the impact of mass transport phenomena on the performance of a bioelectrochemical reactor that was continuously fed with synthetic groundwater containing TCE, the flow rate was changed after 49 days, decreasing it to 2.5 Ld^−1^ and the TCE concentration was kept approximately constant (43.7 ± 1.3 μmolL^−1^). The change of the flow rate (and accordingly of flow velocity) also corresponded to a change in the applied TCE load, which accordingly varied from 63.2 ± 3.6 to 26.3 ± 0.8 μmolL^−1^ d^−1^. This condition has been investigated for 29 days at −650 mV versus SHE cathodic potential applied. After an initial acclimatization period, the RD showed the same trend, in which the TCE is almost completely degraded in the VC intermediate (12.7 ± 1.8 μmolL^−1^) (**Figure** **6**). A lower flow rate applied (2.5 Ld^−1^) resulted in increased amounts of ETH (2.59 ± 0.26 μmolL^−1^) produced during reductive dechlorination. Cis‐DCE was below the detection limit throughout the experiment. Under these conditions, the methane increased (Figure S5, Supporting Information) in average concentration (411 ± 33 μmolL^−1^).^[^ ^21^ ^]^ It showed that the transport of bicarbonate from the bulk liquid to the biofilm was probably limiting the rate of H_2_‐dependent methanogenesis.

The TCE conversion efficiency slightly increased from 80% to around 90% when the flow rate decreased, and VC was the main TCE dechlorination product. The ethene concentration increased when the flow rate was decreased. However, in all conditions, the TCE removal efficiency was almost complete (99%). However, the residual concentration and conversion rate showed a halving when the flow rate was lower, which is consistent with the “lost” products increase. The “lost” term indicates not‐found products in the mass balance on a molar basis, which could also be representative of an oxidative dechlorination of CAHs byproducts to CO_2_. Remarkably, the methane production rate increased much more than the reductive dechlorination rate (up to 1980 μeqL^−1^ d^−1^, at 2.5 Ld^−1^). Consequently, the coulombic efficiency for the RD decreased from 2.43% at 5.5 Ld^−1^ down to 0.55% at 2.5 Ld^−1^. In low‐flow conditions, methanogenesis contributes to more than 30% electron sink. Thus, other competing hydrogen‐consuming microorganisms were stimulated.

### 3.2 Bioelectrochemical Reactor Field Test

**Figure 7:**
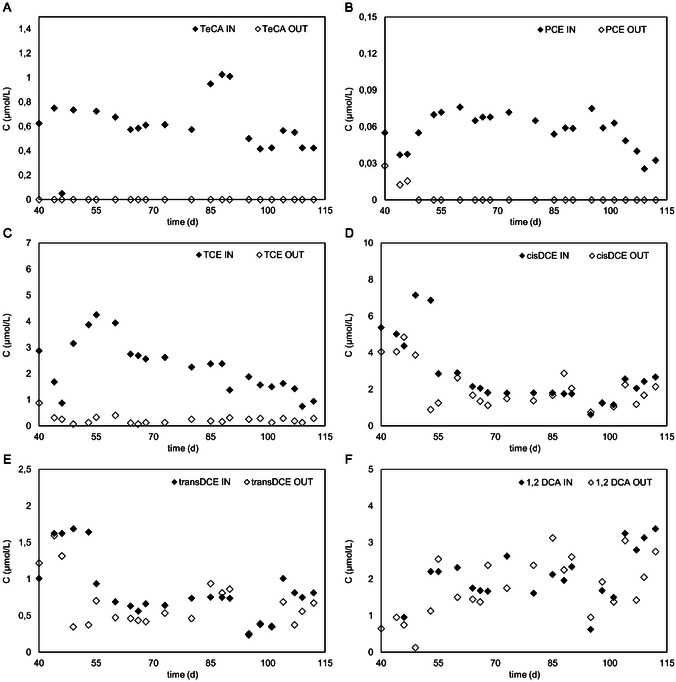
Concentration profile on influent and effluent reactor in the function of time of A) 1,1,2,2‐TeCA, B) PCE, C) TCE, D) cis‐DCE, E) trans‐DCE, and F) 1,2‐DCA.

To prevent the inhibitory effects of low temperatures on biological activity, the field test of the bioelectrochemical reactor was operated with the temperature control system presence to hold biodegradation rates. The temperature was controlled by an electric heater which ensure an almost constant temperature of 15 °C. In the first test stages, the bioelectrochemical reactor was operated continuously in open circuit mode to optimize the system and to inoculate the reactor with indigenous groundwater microorganisms (data not shown). After 40 days of open circuit operation, the cathodic potential of −650 mV versus SHE was applied, resulting in a microbial transformation of higher chlorinated compounds (1,1,2,2‐TeCA, PCE, and TCE) (Figure 10A and 1°C). The main CAHs present in reactor influent and effluent were lower chlorinated RD daughter products (i.e., cis‐DCE, trans‐DCE, and 1,2DCA), while VC, ETH, and ETA were negligible (Figure 10D,E). Figure 1C,D shows the same concentration profile of cis‐DCE and trans‐DCE along the column. However, DCEs decreased by about 30% with residual average concentrations of 1.57 ± 0.13 and 0.50 ± 0.05 μmolL^−1^. 1,2 DCA was almost minimal removal (19%). 1,1,2‐TCA was below the detection limit throughout the experiment. Probably, it represents a rapid stage in the metabolic hydrogenolysis pathway of 1,1,2,2‐TeCA degradation. **Figure** **7**A,B shows a typical profile concentration along the column at this stage at −650 mV versus SHE. 1,1,2,2‐TeCA, PCE, and TCE were almost completely degraded along the column within the second sampling port in the reactive zone (about 100%). Cis‐DCE (Figure 7D) and trans‐DCE (Figure 7E) showed a decreasing trend along the column. DCEs and 1,2‐DCA (Figure 7D) remained constant because they were continually produced and consumed through different metabolic pathways.

**Figure 8:**
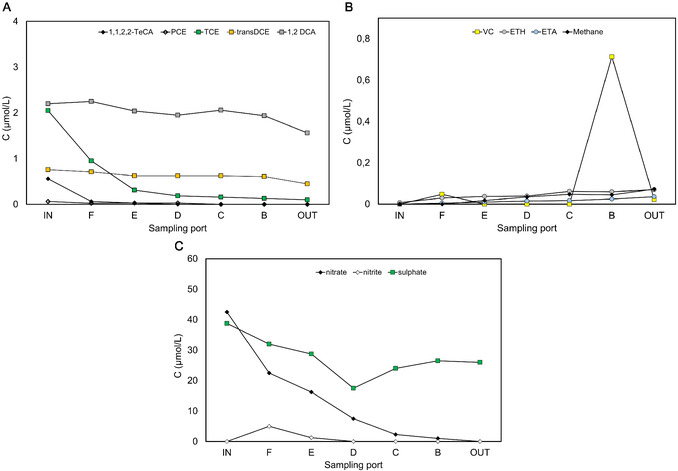
Concentration profile of A) CAHs compound; B) VC, methane, ethylene, and ethane; and C) anion along the column at −650 mV versus SHE.

During the field test, negligible concentrations of VC, ETH, and ETA were produced (**Figure** **8**A,B). The overall concentration of VC, ETH and ETA does not account for 1% of the final total ethenes in the best conditions. In this case, oxidative pathways stimulated by the inner anodic compartment promoted the oxidation of less‐chlorinated daughter products (DCEs and VC). As a result, these compounds were diffusing into the anodic compartment, where they undergo anaerobic oxidative dechlorination, as reported in previous studies.^[^ ^27^, ^28^ ^]^

*Competitive metabolisms* ‐ Methanogenesis was expected to occur after nitrate and sulfate were partially reduced, which showed an H_2_ threshold lower than methanogenesis. Moreover, the methanogen microbes could be inhibited by 1,1,1 TCA, present in groundwater at relatively high concentrations. The methane formation occurred when the 1,1,1 TCA was transformed. After a lag phase, methanogenesis started (Figure 8A) and a high methane concentration (921 ± 59 μmolL^−1^) was reached, suggesting that methanogenesis occurred in the presence of electron acceptors such as sulfate at a lower concentration and in excess of the electron donor.

**Figure 9:**
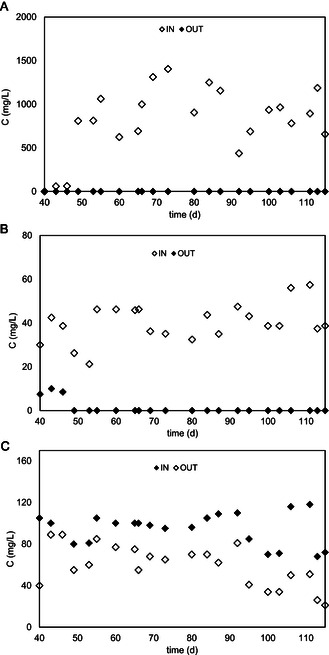
Concentration profile on influent and effluent reactor of A) methane, B) nitrate, and C) sulfate.

The groundwater contained relatively high nitrate (0.6 mmolL^−1^) (**Figure** **9**B) and sulfate (Figure 9C) concentrations (1.1 mmolL^−1^). Those anions are electron acceptors that usually compete with anaerobic dechlorination. Nitrate is the first choice for an electron acceptor in a reducing environment for microbial metabolism. At −650 mV versus SHE, the cathodic potential applied nitrate was expected to promote nitrate reduction by biostimulation of native nitrate‐reducing microbial communities. As shown in Figure 8C, the nitrate concentration was halved at the first sampling port, and only a small transient nitrite concentration was detected between ports F and D; no nitrite and nitrate were detected in the reactor outlet. The sulfate‐reducing reactions removed 33% of sulfate, and it occurred more rapidly until the middle sampling port, where the sulfate is reduced to 38.1 ± 8.5 mgL^−1^. Different from nitrate reduction, the sulfate reduction showed a first reduction step (from port F to port D) and a subsequent oxidation step from port D to the outlet of the reactor; the detected behavior was probably promoted by the presence of the inner anodic compartment (Figure 8C).

The preliminary hydraulic tests, conducted on the laboratory scale, suggested a continuous exchange between the anode and cathode, supporting the hypothesis of oxidation of a low degree of chlorination solvents and reduction/oxidation of sulfate/sulfide along the column. The sulfate suffered a first reduction followed by an oxidation step. The sulfate reduction final product in the cathodic chamber was sulfide, which could have remained in soluble form and been able to diffuse into the anodic chamber, where it could reoxidize to sulfate.^[^ ^23^ ^]^ This latter mechanism may be carried out by many sulfide‐oxidizing microorganisms.^[^ ^29^, ^30^ ^]^ The presence of specific compounds may slow or inhibit cell growth, limiting the CAHs dechlorination. For example, in sites where TCA and TCE are present, TCA can inhibit *Dehalococcoides* from degrading TCE.^[^ ^31^ ^]^ Moreover, the biological degradation pathway of 1,1,1 TCA generally stalls at chloroethane. Figure S5, Supporting Information, shows the trend of 1,1,1 TCA removed percentage as a function of time in the inlet of the reactor. However, the degradation of 1,1,1 TCA occurred until 41% by *Dehalobacter spp.*, which can convert 1,1,1 TCA to chloroethane. Abiotic processes could play a key role in the degradation of TCA to nontoxic end products. The inhibited effect of 1,1,1 TCA was clear for methane formation and sulfate reduction. Moreover, the inhibition of RD was not clear because *Dehalococcoides sp.* has been documented to be inhibited by numerous compounds, i.e., hydrogen sulfide.^[^ ^31^, ^32^, ^33^ ^]^

### 3.3 Electron Balance and Yields of Electron Donor Use

**Figure 10:**
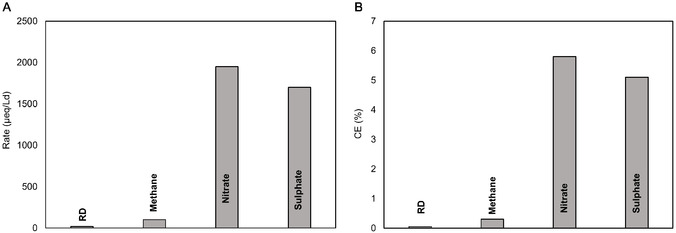
Rate of RD and competitive metabolisms A) and their coulombic efficiency (CE) B).

The overall RD rate (about 5 μeqL^−1^ d^−1^) was calculated by considering the RD products (in terms of reducing equivalent for their formation from the parent compound) (**Figure** **10**A). RD rate remained quite lower than the RD rate observed with the synthetic medium at the same potential (as an example, the RD rate with the TCE synthetic medium was around 157.8 μeqL^−1^ d^−1^ at 2.5 Ld^−1^). This was partially due to different CAHs at different concentrations of groundwater contamination, which synergistically affected microbial dechlorination inhibition.^[^ ^28^ ^]^ The anion reduction reactions occurred at similar rates, 1940 ± 104 μeqL^−1^ d^−1^ and 1772 ± 217 μeqL^−1^ d^−1^ for nitrates and sulfates, respectively. The methanogenesis had a two orders of magnitude lower value (43 ± 3 μeqL^−1^ d^−1^). This is likely due to the greater availability of soluble species like sulfate and nitrate compared to the availability of CO_2_/HCO_3_^−^ for methanogenesis.

The system's overall efficiency was calculated in terms of coulombic efficiency to determine which fraction of the current was used by the different final electron acceptors (Figure 10B). Taking into account all reductive processes (i.e., RD, nitrate and sulfate reductions, methanogenesis, and other unknown metabolisms), the overall coulombic efficiency was 12%.

The lower overall coulombic efficiency is probably derived from the absence of an ionic exchange membrane that promotes an electron loop between the anode and cathode chambers or from an increase in the electrode surface due to the action of oxygen produced in situ on the graphite electrode.^[^ ^18^ ^]^

Therefore, other unknown electron‐consuming mechanisms occurred. This decrease has not been rigorously understood but many factors may influence it. A proposed mechanism could involve a short circuit of hydrogen, which was formed in the cathodic compartment and oxidized in the anodic one, after the diffusion through the separating mesh. This mechanism would represent one of the electrons sink. Moreover, most of the current consumed was used to reduce nitrate (6%) and sulfate (5%). However, RD reached a 0.01% coulombic efficiency.

The efficiency and rate parameters did not consider the synergistic effect of cathodic reduction and anodic oxidation, previously hypothesized, for this purpose has been useful to introduce the CAHs removed percentage parameter (Figure S6, Supporting Information). The spatial variability was considered, so CAHs removed percentages varied along the column. It increased along the column, reaching the maximum at the central sampling port (D), 45%, and subsequently, it remained constant. Therefore, the higher degree of chlorination compounds removal counted more than other CAHs in the overall removal, which decreased as a function of the CAHs concentrations.

### 3.4 Comparison with Existing Approaches and Previous Experience

In BES addressed to bioremediation, the process performances can be performed through the comparison of the coulombic efficiency of the reductive dechlorination reaction. Indeed, this parameter represents the efficiency in supplying electrons to the dechlorinating consortium using the electrodic material instead of conventional fermentative substrates. Despite the low coulombic efficiency (2.43% and 0.01%) for TeCA dechlorination obtained in the present study, those values align with coulombic efficiencies reported for biological reduction of chlorinated hydrocarbons in other experimental investigations.^[^ ^7^, ^28^ ^]^ Low Coulombic efficiencies in BES addressed to CAHs dechlorination are usually recorded due to low contaminant solubility, which affects the reaction rate, while electric current recorded is usually affected by parasitic reactions and possible electrons loop between anode and cathode.^[^ ^17^ ^]^ Despite the low coulombic efficiency, the key role of applied potential and/or fixed current values has been identified as the main factor that allowed reductive and oxidative dechlorination.^[^ ^34^ ^]^ In this way, microbial electrolysis cells can be classified as a primary BES, according to the reference nomenclature reported in the literature.^[^ ^2^ ^]^ As reported in previous studies,^[^ ^4^ ^]^ a case study analysis concerning the utilization of a conventional injection of fermentable substrates to stimulate reductive dechlorination has been performed. The study presents similar efficiencies comparing the information of a case study,^[^ ^23^, ^35^, ^36^, ^37^ ^]^ reporting the use of extra virgin olive oil as fermentable substrate with the typical coulombic efficiency obtained in different BES addressed to chlorinated aliphatic hydrocarbons removal. Some example of literature studies involving the use of BES for CAHs removal in synthetic and real matrices is reported in **Table** **3**

**Table 3:** Literature data about coulombic efficiencies for reductive dechlorination reaction.

Target Compound	BES Configuration	RD Coulombic Efficiency [%]	Reference
PCE	Tubular membrane‐less	22	[17]
PCE–real GW	Tubular membrane‐less	0.013	[28]
PCE—1,2 DCA	Two Chamber/CEM	80.4–90	[36]
*cis*‐DCE	Two Chamber/Nafion	60–90	[23]
TCE‐Cr (VI)	Two Chamber/Nafion	4.66	[37]
TecA	Two Chamber/Nafion	2.6–1.6	[2]

## 4 Conclusions

The cathodic potential applied resulted in a high‐chlorinated CAHs contamination decrease without VC and ETH. The main effect of the potential applied was observed on anion reduction, in which the reaction rates were higher than RD and methane formation. However, the coulombic efficiency reached shallow values (≈10%) due to the overvoltage developed and the hydrogen short circuit along the column. In general, it reached a percentage of about 50% removal.

To realize the bioelectrochemical scaling‐up, several critical points were evaluated. The plant cost was evaluated; for this purpose, no membrane separation between cathodic/anodic zones was present, and the anodic compartment was realized as a separate compartment without continuous flow inside. To limit the overvoltage, the minimal distance and the maximum interface area between the cathode and anode were necessarily realized through an innovative configuration and concentric. However, the anodic oxygen developed could diffuse into the strictly anaerobic cathodic compartment. A sacrificial anode is realized by exploiting the graphite's ability to act as an oxygen scavenger and minimize water electrolysis. The graphite use respects some scaling‐up key points, such as biocompatibility and durability. Electrode materials appear to be resilient under treatment conditions; it is expected that subsurface components of the system can remain effective for 10 or more years. In previous lab‐scale studies, we have demonstrated graphite durability in anodic compartments. In detail, after 5 operating years, the anodic filling showed a similar specific surface area to the unused graphite. This compares favorably against other PRB technologies (e.g., zero‐valent iron), in which PRB materials are consumed. Moreover, the bioelectrochemical reactor showed the main advantage of promoting degradation pathways without chemical dosing and adjusting the potential applied.^[^ ^19^, ^34^ ^]^ Moreover, the possibility of tuning the polarization conditions remotely results in an additional advantage of the technology that allows the creation of reducing and oxidizing conditions in the cathodic and anodic compartment of the reactor, facilitating the transfer of electrons and promoting the transformation of target compounds into nontoxic products.

## Conflict of Interest

The authors declare no conflict of interest.

## Supporting information

Supplementary Material

## Data Availability

The data that support the findings of this study are available from the corresponding author upon reasonable request

## References

[1] M. M. Rossi , E. Dell'armi , L. Lorini , N. Amanat , M. Zeppilli , M. Villano , M. P. Papini , S. Kleinsteuber , B. Matturro , Bioengineering 2021, 8, 109. 34436112 10.3390/bioengineering8080109 PMC8389326

[2] M. Zeppilli , H. Yaqoubi , E. Dell’Armi , A. Lai , M. Belfaquir , L. Lorini , M. P. Papini , Environ. Sci. Ecotechnology 2024, 17, 100309. 10.1016/j.ese.2023.100309 PMC10406622 37560753

[3] B. Matturro , M. Zeppilli , A. Lai , M. Majone , S. Rossetti , Front Microbiol. 2021, 12, 747670. 34659183 10.3389/fmicb.2021.747670 PMC8516407

[4] N. Amanat , B. Matturro , M. Villano , L. Lorini , M. M. Rossi , M. Zeppilli , S. Rossetti , M. Petrangeli Papini , J. Environ. Chem. Eng. 2022, 10, 107047.

[5] A. Marchetti , M. Palhas , M. Villano , J. Fradinho , Catalysts 2024, 14, 239.

[6] F. Marzulli , S. Musivand , M. Arengi , B. De Caprariis , P. De Filippis , A. Marchetti , M. Majone , M. Villano , Chem. Eng. Trans. 2023, 100, 469.

[7] E. Dell’Armi , M. Zeppilli , F. De Santis , M. Petrangeli Papini , M. Majone , ACS Omega 2021, 6, 25211. 34632180 10.1021/acsomega.1c03001 PMC8495709

[8] W. Wang , J. Dong , H. Zhao , Int. Biodeterior. Biodegrad. 2025, 196, 105914.

[9] Y. Yuan , S. J. You , J. N. Zhang , X. B. Gong , X. H. Wang , N. Q. Ren , Environ. Technol. 2015, 36, 1847. 25650667 10.1080/09593330.2015.1013572

[10] B. Kim , J. An , D. Fapyane , I. S. Chang , Bioresour. Technol. 2015, 195, 2. 26122091 10.1016/j.biortech.2015.06.061

[11] E. S. Heidrich , S. R. Edwards , J. Dolfing , S. E. Cotterill , T. P. Curtis , Bioresour. Technol. 2014, 173, 87. 25285764 10.1016/j.biortech.2014.09.083

[12] B. E. Logan , Appl. Microbiol. Biotechnol. 2010, 85, 1665. 20013119 10.1007/s00253-009-2378-9

[13] H. Wang , Z. J. Ren , Biotechnol. Adv. 2013, 31, 1796. 24113213 10.1016/j.biotechadv.2013.10.001

[14] P. Viotti , P. R. Di Palma , F. Aulenta , A. Luciano , G. Mancini , M. P. Papini , Environ. Sci. Pollut. Res. 2014, 21, 1514. 10.1007/s11356-013-2035-9 23933954

[15] F. Aulenta , M. Potalivo , M. Majone , M. P. Papini , V. Tandoi , Biodegradation 2006, 17, 193. 16715399 10.1007/s10532-005-4218-7

[16] Michael. Kasenow , Applied Ground‐Water Hydrology And Well Hydraulics, Water Resources Publications, Highlands Ranch, Colo. 2010.

[17] M. Zeppilli , E. Dell’Armi , L. Cristiani , M. P. Papini , M. Majone , Water 2019, 11, 2579.

[18] A. Lai , F. Aulenta , M. Mingazzini , M. T. Palumbo , M. P. Papini , R. Verdini , M. Majone , Chemosphere 2017, 169, 351. 27886537 10.1016/j.chemosphere.2016.11.072

[19] R. Verdini , F. Aulenta , F. De Tora , A. Lai , M. Majone , Chemosphere 2015, 136, 72. 25950501 10.1016/j.chemosphere.2015.03.092

[20] W. E. Balch , G. E. Fox , L. J. Magrum , C. R. Woese , R. S. Wolfe , Microbiol Rev 1979, 43, 260. 390357 10.1128/mr.43.2.260-296.1979 PMC281474

[21] J. G. Zeikus , Bacteriol Rev 1977, 41, 514. 329834 10.1128/br.41.2.514-541.1977 PMC414011

[22] G. Sassetto , L. Lorini , A. Lai , M. Petrangeli Papini , M. Zeppilli , Catalysts 2024, 14, 208.

[23] A. Lai , R. Verdini , F. Aulenta , M. Majone , Chemosphere 2015, 125, 147. 25556008 10.1016/j.chemosphere.2014.12.023

[24] J. M. Gossett , Environ Sci Technol 1987, 21, 202.

[25] A. Di Battista , R. Verdini , S. Rossetti , B. Pietrangeli , M. Majone , F. Aulenta , N. Biotechnol. 2012, 30, 33. 22728722 10.1016/j.nbt.2012.06.002

[26] M. Zeppilli , B. Matturro , E. Dell’Armi , L. Cristiani , M. P. Papini , S. Rossetti , M. Majone , J. Environ. Chem. Eng. 2021, 9, 104657.

[27] M. Zeppilli , E. Dell'armi , M. P. Papini , M. Majone , Chem. Eng. Trans. 2021, 2021.

[28] E. Dell’Armi , M. Zeppilli , M. L. Di Franca , B. Matturro , V. Feigl , M. Molnár , Z. Berkl , I. Németh , H. Yaqoubi , S. Rossetti , M. P. Papini , M. Majone , J. Water Process Eng. 2022, 49, 103101.

[29] K. Fuseler , H. Cypionka , Arch Microbiol 1995, 164, 104.

[30] K. Fuseler , D. Krekeler , U. Sydow , H. Cypionka , FEMS Microbiol. Lett. 1996, 144, 129.

[31] M. Duhamel , S. D. Wehr , L. Yu , H. Rizvi , D. Seepersad , S. Dworatzek , E. E. Cox , E. A. Edwards , Water Res. 2002, 36, 4193. 12420924 10.1016/s0043-1354(02)00151-3

[32] J. He , Y. Sung , R. Krajmalnik‐Brown , K. M. Ritalahti , F. E. Löffler , Environ. Microbiol. 2005, 7, 1442. 16104866 10.1111/j.1462-2920.2005.00830.x

[33] D. T. Adamson , G. F. Parkin , Environ. Sci. Technol. 2000, 34, 1959.

[34] F. Aulenta , L. Tocca , R. Verdini , P. Reale , M. Majone , Environ Sci Technol 2011, 45, 8444. 21877695 10.1021/es202262y

[35] C. C. Azubuike , C. B. Chikere , G. C. Okpokwasili , World J. Microbiol. Biotechnol. 2016, 32, 180. 27638318 10.1007/s11274-016-2137-x PMC5026719

[36] F. Chen , B. Liang , Z. L. Li , J. Q. Yang , C. Huang , M. Lyu , Y. Yuan , J. Nan , A. J. Wang , Chemosphere 2019, 227, 514. 31004818 10.1016/j.chemosphere.2019.04.066

[37] A. Lai , M. L. Astolfi , V. Bertelli , V. G. Agostinelli , M. Zeppilli , M. Majone , New Biotechnol. 2020. 10.1016/j.nbt.2020.06.006 32683048

