# Supplementary material for: Field Test of a Bioelectrochemical Membrane‐Less Reactor for Chlorinated Aliphatic Hydrocarbon and Nitrate Removal from a Contaminated Groundwater
Source: Chempluschem. 2025 Jun 16;90(8):e202400683. doi: 10.1002/cplu.202400683 (PMC12352736; doi:10.1002/cplu.202400683)
Supplement: Supplementary file 1 — Supplementary Material [file CPLU-90-e202400683-s001.pdf]

Supplementary Material

Field Test of a Bioelectrochemical Membrane-Less Reactor for Chlorinated Aliphatic Hydrocarbon and Nitrate Removal From a Contaminated Groundwater

Geremia Sassetto[a], Maria Presutti[a], Agnese Lai[a], Giulia Simonetti[a], Laura Lorini[a], Marco Petrangeli Papini[a] and Marco Zeppilli*[a, b]

[a] Department of Chemistry
Institution: University of Rome Sapienza
Address: Piazzale Aldo Moro 5, 00185 Rome
E-mail: (marco.zeppilli@uniroma1.it)

[b] Research Center for Applied Sciences to the Safeguard of Environment and Cultural Heritage (CIABC)
Institution: University of Rome Sapienza
Address: Piazzale Aldo Moro 5, 00185 Rome

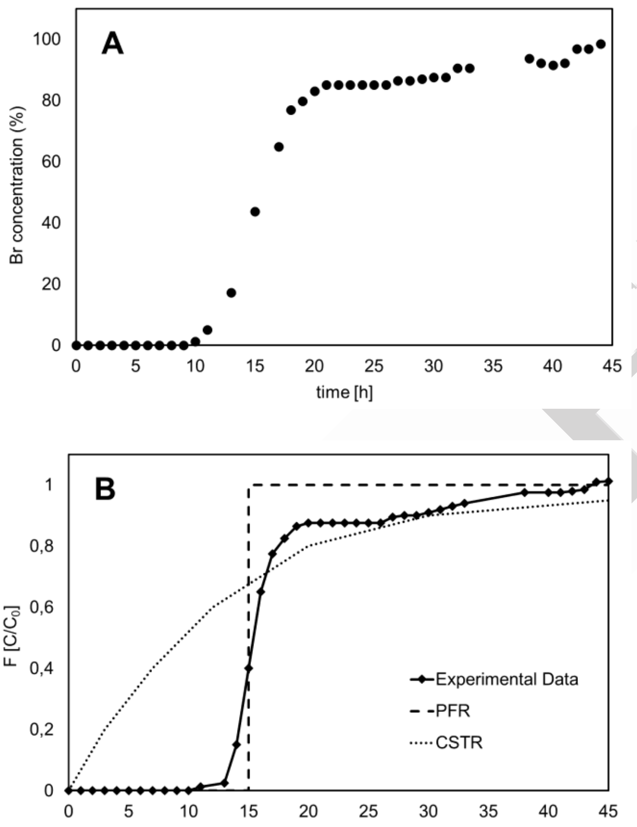

Figure S1. (A) Tracer step experiment bromide % concentration and (B) experimental residence time distribution curve F(t) and the theoretical perfect mixing flow (CSTR) and plug flow (PFR) RTD curves in reactor output (sampling port B).

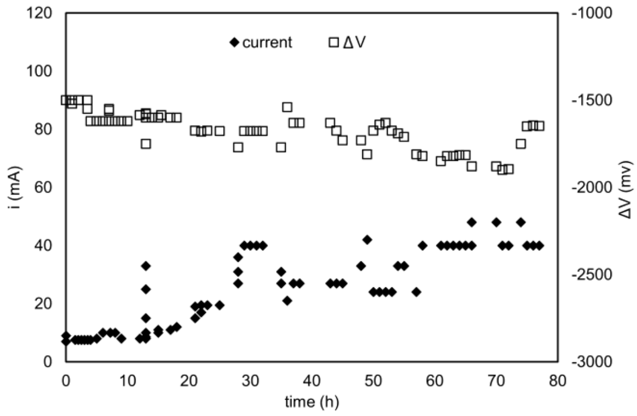

Figure S2. Cell voltage and current of the pilot-scale BES with cathodic potential applied -650 mV vs SHE during the start-up period.

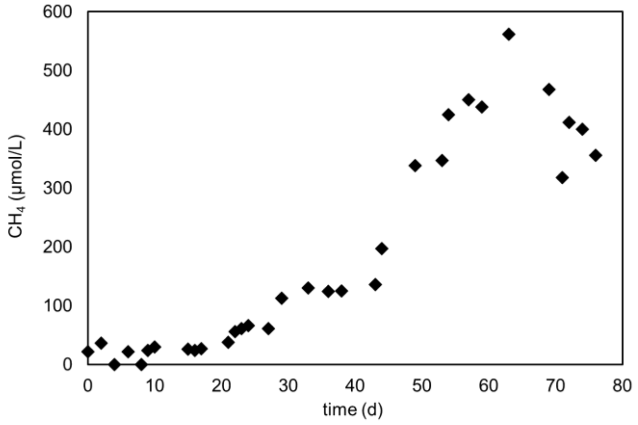

Figure S3. Methane concentration profile as a function of time during the reactor operation at

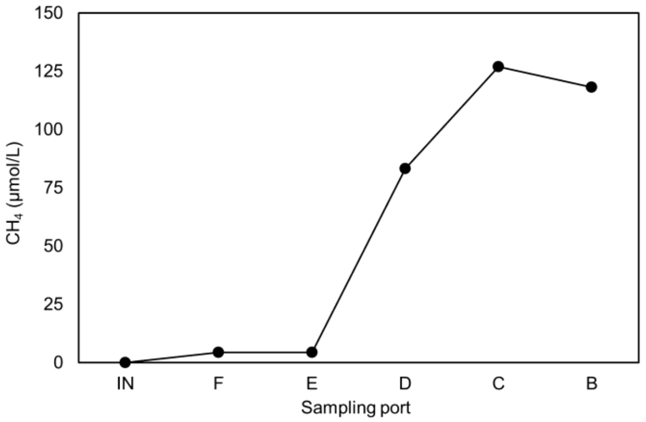

Figure S4. Methane concentration profile in function on column length at 5.5 Ld^{-1}.

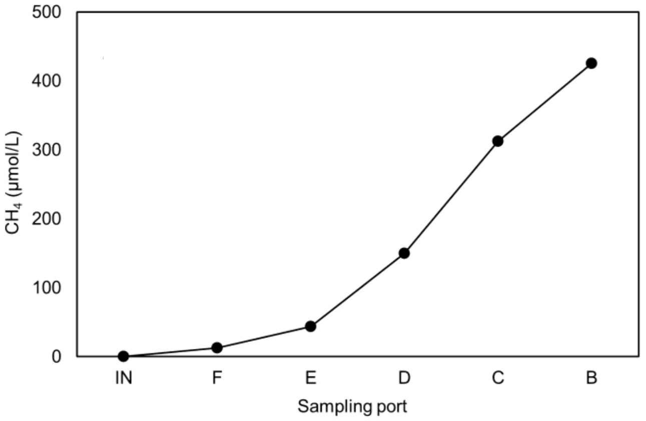

Figure S5. Methane concentration profile in function on column length at 2.5 Ld^{-1}.

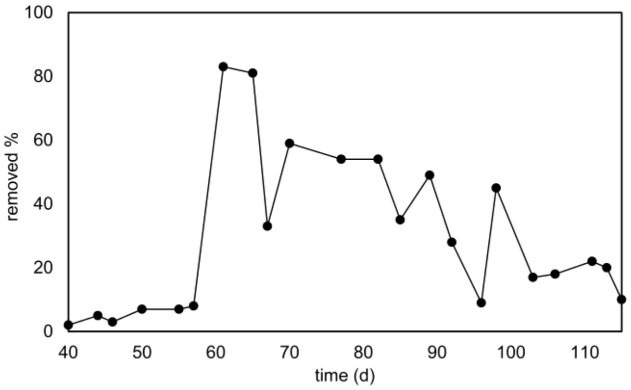

Figure S6. Removed percentage of 1,1,1 TCA during the . 2.5 Ld^{-1} operating period.

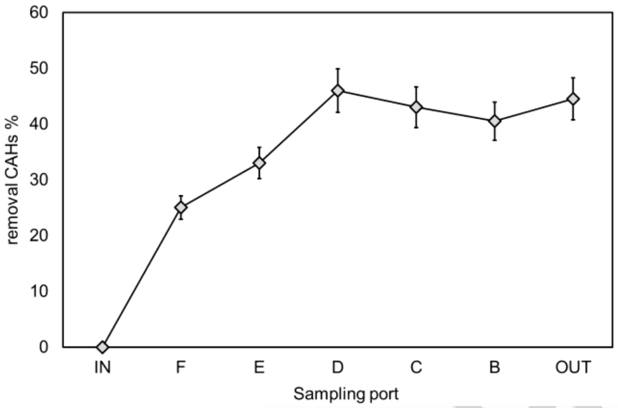

Figure S7. Average CAHs removal percentage obtained during the different operating conditions as a function of column length.
